# Clinical Actionability of the Genomic Landscape of Metastatic Castration Resistant Prostate Cancer

**DOI:** 10.3390/cells9112494

**Published:** 2020-11-17

**Authors:** Wout Devlies, Markus Eckstein, Alessia Cimadamore, Gaëtan Devos, Lisa Moris, Thomas Van den Broeck, Rodolfo Montironi, Steven Joniau, Frank Claessens, Thomas Gevaert

**Affiliations:** 1 Department of Urology, University Hospitals Leuven, 3000 Leuven, Belgium; gaetan.devos@uzleuven.be (G.D.); lisa.moris@uzleuven.be (L.M.); thomas.vandenbroeck@uzleuven.be (T.V.d.B.); steven.joniau@uzleuven.be (S.J.); 2 Laboratory of Molecular Endocrinology, KU Leuven, 3000 Leuven, Belgium; frank.claessens@kuleuven.be; 3 Department of Pathology, Friedrich-Alexander-University of Erlangen-Nürnberg, 91054 Erlangen, Germany; markus.eckstein@uk-erlangen.de; 4 Section of Pathological Anatomy, School of Medicine, Polytechnic University of the Marche Region, United Hospitals, 60121 Ancona, Italy; a.cimadamore@staff.univpm.it (A.C.); r.montironi@staff.univpm.it (R.M.); 5 Department of Pathology, University Hospitals Leuven, 3000 Leuven, Belgium; thomas.gevaert@kuleuven.be

**Keywords:** prostate cancer, biomarker, targeted therapies, histology, mCRPC, androgen receptor

## Abstract

The development of targeted therapies increases treatment options for metastatic castration resistant prostate cancer (mCRPC) patients. There is a need for strong predictive and prognostic signatures to guide physicians in treating mCRPC patients. In this review we unravel the possible actionability in the AR pathway, PI3K AKT signaling, and DNA repair pathways. Additionally, we make recommendations on biomarker trial design, and the clinical use of this new type of data.

## 1. Introduction

**Figure 1:**
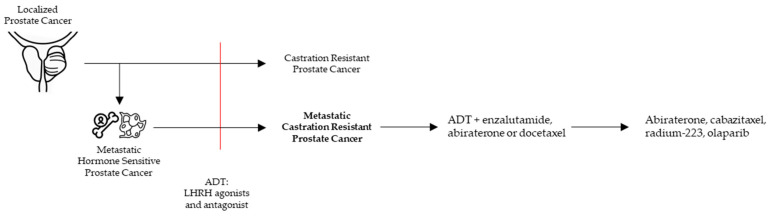
Clinical pathway to metastatic castration resistant prostate cancer (mCRPC) with the current treatment options.

Prostate cancer (PCa) is not a uniform disease as its outcome is highly associated with the corresponding clinical risk category. In general, PCa is strongly driven by androgen receptor (AR) regulated transcription [1]. However, PCa can become ‘castration resistant’ (CRPC) after escaping conventional androgen deprivation treatment (ADT, LHRH agonists, and antagonists). As prolonged and intense targeting of the AR in CRPC patients resulted in a survival benefit, ‘castration resistance’ often remains AR driven (Figure 1).

Metastatic castration resistant prostate cancer (mCRPC) results from many genomic and transcriptomic changes. The heterogeneous appearance of these changes underly the multifocal nature of PCa. It is thought that AR insensitive clones will survive the therapeutic selection pressure by AR targeting treatments. As mCRPC patients have a bad prognosis, these patients need to be rapidly treated with drugs likely to be effective. Identifying patients that would benefit from specific treatments will need to be based on specific markers, which could be detected as genomic and/or transcriptomic signatures.

In this review we will look at these genomic and transcriptomic alterations, with particular attention on those with prognostic and predictive potential. 

## 2. mCRPC in the Clinic

### 2.1. Approved Treatments in Clinic

CRPC is diagnosed in PCa patients with castrate serum testosterone < 50 ng/dL and biochemical or radiological progression [2] (Figure 1). mCRPC is a noncurable, fatal disease with a median overall survival of 2.8 years [3,4]. In general, mCRPC patients will receive multiple lines of therapies. Accepted therapies available in the clinic can largely be classified in AR signaling inhibitors (ARSI), chemotherapeutics, and the alpha emitter radium-223. ARSI result in a strong inhibition of the AR pathway by drugs targeting the androgen synthesis (abiraterone) and antagonists of the AR (enzalutamide, darolutamide, apalutamide). Chemotherapeutics consist of tubulin binding docetaxel and the 2nd generation cabazitaxel (Figure 1).

Besides the standard of care, novel targeted therapies are entering clinic with PARP (poly-ADP ribose polymerase)-inhibitors being beneficial in PCa escaping from ARSI treatment on the condition that homologous recombination repair (HRR) alterations are present [5]. Other promising targeted therapies are still investigational and will be discussed throughout this review.

### 2.2. Treatment Allocation and Decision Making

It has been shown that the overall survival gain of consecutive treatments is not equal to the survival gain of each drug separately [3]. Combination treatments are thought to be superior over sequential treatments [6].

Most recent guidelines advise to base the choice of first-line treatment on different patient characteristics including performance status, symptoms, comorbidities, location and extent of disease, patient preference, and previous treatments [2]. The optimal treatment sequence, however, remains unclear. This contrasts the need for rapid and adequate treatment in mCRPC patients. Improvements in the understanding of PCa biology should help clinicians to decide on the optimal treatment sequence.

### 2.3. Role of Genetic Sequencing

In mCRPC, genetic sequencing on different time point can guide the management throughout the course of the disease [7,8]. Although sequencing of metastatic lesions reveals the main oncogenic drivers, obtaining this tissue can be practically challenging [9]. Sequencing circulating tumor cells or DNA isolated from liquid biopsies offers practical alternatives, as their mutations possibly reflect genomic alterations in the tissue [8].

TCGA, a molecular classification initiative, aimed to classify different tumors, including prostate cancer, by their genomic alterations. In mCRPC, large sequencing initiatives identified the genomic alterations and confirmed possible actionable targets to be present in most patients [10,11,12]. As discussed further, some alterations will predict a good response to drugs already available in the clinic, while others require the development of novel targeted therapies.

Studies confirmed a possible benefit of using genomic information to help treatment allocation [5,13,14]. However, as PCa is heterogenous, more clinical studies are needed to define the contribution of genomic information to the management of mCRPC patients.

## 3. PCa Biology and Markers

### 3.1. Genomic and Transcriptomic Pathways in PCa 

#### 3.1.1. AR Pathway

The AR signaling pathway is essential for prostate development and throughout different stages of PCa [1]. As ADT and/or ARSI are administered before patients develop mCRPC, AR pathways are altered in about 70% of the mCRPC cases [10]. These alterations can result in both AR dependent and independent disease [15].

The AR dependent resistance mechanisms cover AR overexpression, AR gene amplifications, mutations, splice variants, and alterations of coregulators, with the majority being amplifications. Amplifications result in an increased sensitivity to androgens due to an overexpression of AR [10]. In another study more than 70% of mCRPC patients had duplications of an upstream enhancer of AR, suggesting this to be a causative step in the development to CRPC [16].

AR mutations are most often found in the Ligand Binding Domain (LBD) [10]. These mutations are mostly situated within the ligand binding pocket, resulting in altered hormone binding and an altered response to AR targeting. Mutations L702H, T878A, and H875Y were associated with poor response to abiraterone, while enzalutamide and apalutamide act as agonists for the F877L and F876L mutated AR [17,18,19]. The agonistic effect of anti-androgens on the F877L mutation is found to be more apparent when comutated with the T878A mutation [20]. Darolutamide, a novel ARSI, maintains its antagonistic characteristics in F876L mutated PCa [21]. Taken together, clinical PCa treatment could be guided by the presence of AR mutations.

Although still strongly debated, expression of splice variants, e.g., AR-V7, Arv567es, may cause the PCa cells to become insensitive to ADT or ARSI [22]. AR-V7 has been proposed as clear prognostic factor, yet its detection by immunohistochemistry or qPCR needs optimization before routine application. Once this is available, it will be possible to assess the true value of functional AR-V7 status [22,23]. As these AR splice variants all change or lose the ligand binding domain of the AR, classic therapies targeting this region lose their effectiveness [24,25]. Yet, splice variants are always coexpressed with high levels of full-length AR. Additionally, these splice variants interact differently with the microtubular structure of the cell, rendering them more resistant to taxane chemotherapeutics [6,26]. The anti-helminthic drug niclosamide is under clinical investigation as it led to degradation of AR-V7, and resulted in a reappearance of sensitivity to anti-androgens in preclinical settings [6,27,28].

Different coregulators have been described to be involved in the AR axis, including: NCOA1, 2, 3, p300, transcription factor FOXA1, negative regulator NCOR1/2, and AR induced negative regulator ZBTB16 [10,29]. Preclinical p300 inhibitors and investigational inhibitory strategies for the coactivators are available and show benefit in preclinical settings [30]. Interestingly, these coregulators are used by different families of transcription factors, making their PCa specific targeting challenging. Possible clinical benefits of targeting the coregulators remain to be discovered.

SPOP is described as tumor suppressor gene, degrading oncogenic substrates like NCOA3/SRC3 (co-activator of AR), ERG, and DEK [31,32]. Besides this, SPOP is also involved in ubiquitination and degradation of the AR [33]. In PCa SPOP is often mutated leading to increased androgen signaling and tumor progression [34,35]. Consequently, these tumors are highly AR regulated and form a distinct cluster in the TCGA molecular classification [13]. In the localized setting SPOP mutations are the most frequent alteration (13%), but it is less frequently found in the metastatic cohort (around 8%) [10,36,37]. In the later stages of PCa, SPOP mutations have therapeutic implications, being more resistant to chemotherapeutics and more susceptible for AR targeting [38,39]. Interestingly SPOP mutations are mutually exclusive with changes in TP53, PTEN, or TMPRSS2-ERG [37].

Erythroblast Transformation Specific (*ETS*) gene fusions are the most common genetic alteration in cancer and it is found in approximately 50% of localized PCa as well as in mCRPC [10,40]. Fusions link the transcriptional control regions of a prostate-specific, AR regulated, *TMPRSS2* gene to the protein coding part of members of the oncogenic *ETS* gene family (e.g., *ERG*). This renders the *ETS* oncogene family AR dependent and highly expressed. *TMPRSS2-ERG* fusions are more resistant to treatment with taxane-chemotherapeutics and, as they are AR regulated, these patients are more susceptible to ADT and ARSI [41,42,43].

A lot of attention has been attributed to glucocorticoid receptor (GR) upregulation as escape mechanism after ARSI treatment. As GR and AR have very similar mechanisms of action, the GR can take over part of the AR pathway [44]. Moreover, in neoadjuvant ARSI trials, GR expression and activity are correlated with higher residual tumor volume in different studies based on immunohistochemistry and transcriptome changes [45,46].

GR antagonists were found to be beneficial as adjuvant treatment to ARSI in preclinical models, as the expression of GR and AR seemed to be inversely correlated [47]. Androgens and glucocorticoids are known to affect each other’s signaling pathways, which suggests both pathways should be targeted in order to be effective [48]. In different clinical mCRPC treatment regiments (chemotherapeutics and abiraterone) glucocorticoids are coadministered to diminish side effects, possibly stimulating GR upregulated tumors to progress [47]. A phase II trial investigated the use of the GR-antagonist mifepristone monotherapy as an AR antagonist in 19 both non-metastatic and metastatic CRPC patients [49]. GR blockage resulted in upregulated circulating androgens due to a feedback via adrenocorticotropic hormone (ACTH) inducing adrenal androgens and their conversion to testosterone and DHT. This feedback likely masked the therapeutic value of mifepristone in CRPC patients. It will therefore be interesting to see the effect of combined treatment with ARSI and GR antagonists, currently under investigation in a phase I/II trial [50].

Castration and abiraterone both target the AR axis by depleting its ligands, but this inhibition is overcome by the activation of the tumoral steroid synthesis. Cancer cells of local and metastatic disease can synthesize DHT from adrenal precursors, resulting in a release of the inhibited AR [51]. For example, the 3β-hydroxysteroid dehydrogenase isoenzyme-1 (HSD3B1) can become expressed in these cells and will convert dehydroepiandrosterone (DHEA) to androstenedione and androstenediol to testosterone. ARSI treatments induce HSD3B1 levels, by decreasing proteasomal degradation. Interestingly, single nucleotide polymorphisms in the *HSD3B1* gene also affect the expression levels. This results in higher concentrations of androstenedione, and therefore DHT [52,53]. Preclinical models are moreover suggestive that some adrenal steroidogenesis remains upon CYP17A inhibition, by proving that adrenalectomy has stronger effects than CYP17A inhibition [54]. Further translational studies should try to target these escape mechanisms, in order to exploit this new knowledge clinically.

In conclusion, as PCa is an androgen driven tumor, different escape mechanisms alter the AR pathway. Future efforts should not only be directed to the characterization of AR alterations and common other AR escape mechanisms, but also focus on targeting the steroid metabolism and the GR pathway.

#### 3.1.2. PI3K–AKT–MAPK Pathway—PTEN Loss

As *PTEN* is a major regulator of the cell cycle and tumor suppressor gene, its loss is associated with poor clinical outcome and progression to mCRPC [55,56,57,58,59]. Deletion of PTEN is more often present in mCRPC (17% in localized and 40% of mCRPC cases), independent of metastatic load [10,36,60]. In mCRPC, *PTEN* loss is associated with *ETS* rearrangements (see above), enforcing their mutagenic capacities [61,62].

The exact mechanisms explaining how PTEN leads to castration resistance are still debated. The inhibition of the PI 3K pathway (PI3K, AKT, mTORC1/2), via AKT inhibition by PTEN, is considered an important contributor [63]. As AKT promotes cell survival and its activation is associated with multiple cancers, AKT inhibitors have been developed [63]. Preclinical evidence in *PTEN* deleted models showed lower AR activity after activation of the PI 3K pathway. As AR and PI 3K compensate for each other’s inhibition, a dual inhibition of both pathways, consisting of an AKT inhibitor and an ARSI, seems promising [64]. A phase III trial studying this dual inhibition (ipatasertib/abiraterone) is currently running in mCRPC patients with *PTEN* loss [65].

#### 3.1.3. DNA Repair

Of all germline variants found in metastatic cancers, 75% were related to defects in DNA repair confirming the importance of aberrant DNA repair in carcinogenesis [66]. Although localized PCa has a low mutational burden, germline mutations in DNA repair genes are present in 12% of mCRPC patients [66,67]. During synthesis (S) or Gap 2 (G2) phase homologous recombination repair (HRR) accurately repairs double strand breaks. Whenever the HRR pathway is deficient, other repair pathways take over to repair DNA damage, albeit all with higher failure rates. Most common genetic mutations affecting HRR are BRCA2, CHEK2, ATM, and BRCA1 [5,68].

Mutations in HRR render patients sensitive to PARP inhibitors (PARPi), as PARP is involved in the repair of single strand DNA breaks. If unrepaired, these are transformed to double strand DNA breaks during replication. PARPi are therefore called synthetically lethal because these are only effective when repair mechanisms fail [5,68]. This explains why PARPi would preferentially target tumor cells, contributing to the favorable risk profile. The phase III PROfound trial studied PARPi in 387 progressive mCRPC patients, with confirmed prolonged progression free survival and good functional outcomes in patients with HRR defects [5].

Mismatch repair (MMR) genes will correct nucleotide allocation mistakes by an excision and reallocation of the base by verifying the genetic code on the adjacent strand. Different genes have been described to be involved in MMR, including: *MLH1*, *MLH3*, *MSH2*, *MSH3*, *MSH6, PMS1,* and *PMS2*. Tumors with MMR mutations typically accumulate large numbers of mutations and microsatellite instability. Such tumors can be relatively easily identified with immunohistochemistry (see below) and have been shown to respond well to both ADT and ARSI treatment and possibly immune checkpoint inhibitors [69,70].

Cyclin dependent kinase 12 (CDK12) is a member of the kinases, involved in the regulation of the cell cycle. Biallelic loss of *CDK12* defines a distinct subtype of PCa and is present in 5% of mCRPC [10]. When present, most patients have no alterations in genes involved in other oncogenic pathways like *ERG*, *SPOP*, *TP53*, and *PTEN* [71]. Tumors with *CDK12* loss show aberrant DNA replication during S-phase and have a high immune infiltration [72]. A retrospective multicenter study suggested *CDK12* loss to form an aggressive PCa subtype with poor responses to hormonal and taxane treatment as well as PARPi. Surprisingly, good response of this subtype was seen after treatment with immune checkpoint inhibitors, possibly linked to their higher mutation burden [71]. Further prospective studies should be performed to investigate the underlying biology as well as the opportunities in clinical management of these patients.

#### 3.1.4. Neuroendocrine Differentiation

In this review we focused on adenocarcinoma of the prostate. Although this is not the only histopathologic form of mCRPC, it covers more than 95% of all mCRPC cases [10]. Preclinical evidence suggests neuroendocrine features to be selected during AR targeting treatments or via an accrual of mutations [73,74]. Different gene sets have been developed to assess neuroendocrine transdifferentiation, yet these should be validated in the mCRPC setting [75].

In mCRPC patients it is unclear what percentage of tumors develop into a neuroendocrine state, and what percentage result from a neuroendocrine population being positively selected as a result of the treatments. The latter has enriched losses of p53 and Rb1 and should be treated early on [76,77].

**Figure 2:**
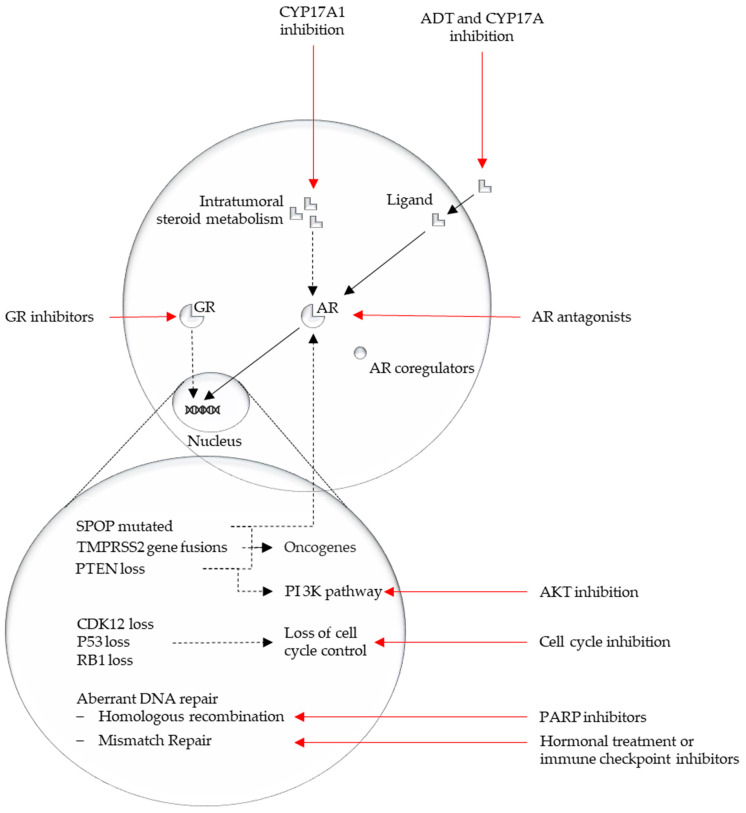
Overview major affected pathways in mCRPC and their possible actionability.

An overview of the discussed pathways from this section is represented in Figure 2.

### 3.2. Biomarkers

A good biomarker has reproducible predictive or prognostic characteristics in a well-defined disease state, and provides information that can impact the treatment allocation and the further care. As a biomarker should be reproducible in a certain disease state, different patients, and trial settings, a reliable predictive risk profile will most likely consist of different biomarkers. Here, we focus on the possible contribution of genomic biomarkers in future PCa care.

Although there are many reports suggesting new biomarkers for different specific PCa settings, limited clinical improvements have been made [78,79]. One approach using the adapted genomic PAM50 classifier from breast cancer can classify PCa into luminal and basal subtypes [80]. AR expression is higher in luminal than basal subtypes. Furthermore, luminal B subtype was proven to benefit from additional ADT after primary treatment [81]. Luminal A subtypes had the best survival and overall, the luminal subtypes were more sensitive than the basal subtype to docetaxel and less sensitive to platinum and etoposide chemotherapeutics [82].

Histopathology is an elegant way to detect protein-biomarkers. Firstly, although hematoxylin-eosin staining suffices to find malignant cells, some markers (like AR) can help to reduce doubt [83]. Second, some histological entities (e.g., cribriform PCa) are associated with molecular alterations (e.g., *PTEN* loss, *SPOP,* and *ATM* mutant) and can therefore reflect corresponding underlying biomarkers [84]. Lastly, histological markers reflect underlying transcriptomic or genomic alterations and can therefore often predict outcomes accordingly. A big advantage of immunohistochemistry to check for underlying biomarkers is that this methodology and corresponding antibodies are readily available and mostly rather cheap (e.g., AR targeted treatment: *PTEN* and *ERG*) [59].

### 3.3. Prospects

Most candidate biomarkers result from small, hypothesis generating, studies. Subsequent biomarker trials should therefore be run to validate them for specific indications. Besides treatment allocation, signatures could identify reasons for treatment intensification, drug selection, and duration of therapy. In further disease stages the role in defining the treatment-sequence becomes equally important [14]. Good examples of upcoming treatment allocation trials are the GUNS and the ProBio trial. The GUNS trial will investigate the guided treatment allocation using gene signatures from a prostate biopsy [85]. In mCRPC patients, the ProBio trial uses cfDNA in mCRPC patients to allocate them to treatment arms based upon genomic alterations [86].

Multiple attempts have already been done to categorize PCa based on its molecular characteristics, with the TCGA being the most promising [13,79,87]. However, in a study where each PCa tumor focus was sequenced, only 30% of patients could be classified in one of the proposed classes [88]. Furthermore, a preclinical actionable target is present in almost 90% of mCRPC patients, but this cannot be implemented into clinic due to the lack of specific inhibitors and high-quality clinical trials [10].

**Figure 3:**
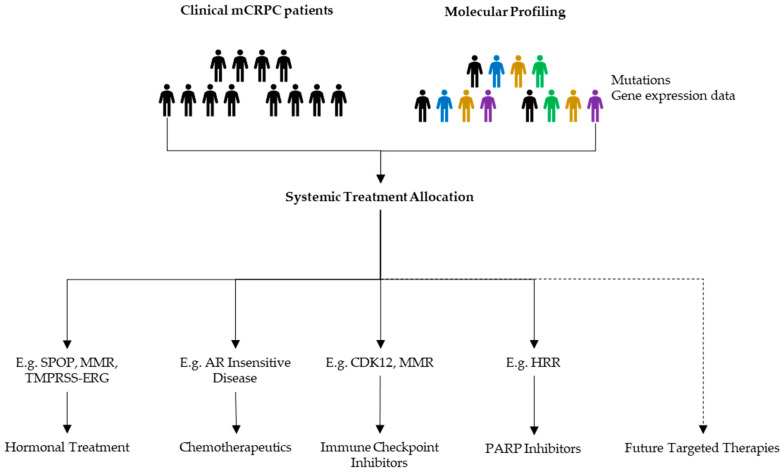
Possible benefits of molecular profiling in mCRPC management. When progressive disease appears, discussions upon next line treatments should be based upon all available data to allocate a patient tailored follow-up treatment.

Besides genomic alterations, which were the topic of this review, there are multiple clinical biomarkers being studied. The overarching aim is that some of these factors will contribute to the identification of specific patient profiles that will allow clinicians to propose treatments likely to be effective (Figure 3).

Additionally, by increasing knowledge on cancer biology throughout the following years, novel treatment options that are not included in this review will be suggested and investigated. These can be targeted towards mCRPC or act as an adjuvant treatment to block certain escape mechanisms.

## 4. Conclusions

To increase PCa survival, multidisciplinary oncological PCa boards should be able to use all available diagnostic data for their discussions and treatment decisions (Figure 3). These data should include clinical characteristics, imaging, and histology. Additional predictive or prognostic biomarker-profiles based on molecular and genomic information may help to guide the clinical team towards the optimal treatment for each mCRPC patient.

Defining better subclassifications of mCRPC populations that respond well to given treatments is therefore a high priority in mCRPC care. Predictive molecular biomarkers for the treatment of mCRPC are intensively investigated, but until now, only limited clinical implementation has been realized.
